# State-of-the-Art Evidence Retriever for Precision Medicine: Algorithm Development and Validation

**DOI:** 10.2196/40743

**Published:** 2022-12-15

**Authors:** Qiao Jin, Chuanqi Tan, Mosha Chen, Ming Yan, Ningyu Zhang, Songfang Huang, Xiaozhong Liu

**Affiliations:** 1 Alibaba Group Hangzhou China; 2 Zhejiang University Zhejiang China; 3 Indiana University Bloomington Bloomington, IN United States

**Keywords:** precision medicine, evidence-based medicine, information retrieval, active learning, pretrained language models, digital health intervention, data retrieval, big data, algorithm development

## Abstract

**Background:**

Under the paradigm of precision medicine (PM), patients with the same disease can receive different personalized therapies according to their clinical and genetic features. These therapies are determined by the totality of all available clinical evidence, including results from case reports, clinical trials, and systematic reviews. However, it is increasingly difficult for physicians to find such evidence from scientific publications, whose size is growing at an unprecedented pace.

**Objective:**

In this work, we propose the PM-Search system to facilitate the retrieval of clinical literature that contains critical evidence for or against giving specific therapies to certain cancer patients.

**Methods:**

The PM-Search system combines a baseline retriever that selects document candidates at a large scale and an evidence reranker that finely reorders the candidates based on their evidence quality. The baseline retriever uses query expansion and keyword matching with the ElasticSearch retrieval engine, and the evidence reranker fits pretrained language models to expert annotations that are derived from an active learning strategy.

**Results:**

The PM-Search system achieved the best performance in the retrieval of high-quality clinical evidence at the Text Retrieval Conference PM Track 2020, outperforming the second-ranking systems by large margins (0.4780 vs 0.4238 for standard normalized discounted cumulative gain at rank 30 and 0.4519 vs 0.4193 for exponential normalized discounted cumulative gain at rank 30).

**Conclusions:**

We present PM-Search, a state-of-the-art search engine to assist the practicing of evidence-based PM. PM-Search uses a novel Bidirectional Encoder Representations from Transformers for Biomedical Text Mining–based active learning strategy that models evidence quality and improves the model performance. Our analyses show that evidence quality is a distinct aspect from general relevance, and specific modeling of evidence quality beyond general relevance is required for a PM search engine.

## Introduction

Traditionally, patients with the same diseases are treated with the same therapies. However, the treatment effects can be highly heterogeneous, that is, the benefits and risks may differ substantially among patient subgroups [1]. The precision medicine (PM) research initiative [2] takes into account individual differences in people’s genes, environments, and lifestyles when tailoring their treatment and prevention strategies. Under the ideal paradigm of PM, patients of the same diseases are divided into several subgroups, and different patient subgroups receive different treatments that are the most suitable for them. PM is now widely applied in oncology, since sequencing techniques can identify considerable genetic variations in patients with cancer. For example, patients with non–small cell lung cancer with epidermal growth factor receptor gene mutations are sensitive to gefitinib therapy [3], and patients with breast cancer who have human epidermal growth factor receptor 2 mutations are sensitive to trastuzumab therapy [4].

PM practices should be guided by the principles of evidence-based medicine [5], where treatments are based on high-quality clinical evidence, such as systematic reviews and randomized controlled trials, instead of individual experiences. However, as the number of scientific publications is growing rapidly (eg, about 2700 articles are added to PubMed each day in 2019), it is difficult for physicians to find clinical evidence in the literature that supports or reject specific treatment options for certain patients. Information retrieval (IR) is aimed at automatically finding relevant documents for users’ queries. IR has been successfully applied to the general consumer and biomedical research domain with search engines such as Google and PubMed. However, most current search engines cannot process PM queries that contain structured information about patients and therapies and neither do they rank the documents based on their significance as clinical evidence.

To facilitate IR research for PM, the Text Retrieval Conference (TREC) holds the PM Track annually since 2017. From 2017 to 2019, the TREC PM focused on finding relevant academic papers or clinical trials of patient topics specified by their demographics, diseases, and gene mutations [6-8]. In 2020, the TREC PM focus was changed to retrieve academic papers that report critical clinical evidence for or against a given treatment in a population specified by its disease and gene mutation [9]. Both supporting and opposing clinical evidence are important, because they provide valuable guidance to clinical decision making regarding whether or not to use the treatment. To assist the practices of PM, such as in the case of the TREC PM task, the most vital property of a retriever is to rank the relevant papers by their evidence quality, that is, to what extent they can assist clinical decision-making. The objective of this work was to develop a retrieval model that can rank relevant papers by their evidence quality to a given PM topic.

Traditional IR systems are mostly based on term frequency–inverse document frequency and its derivatives that basically rank the documents by their bag-of-word similarities with the input query. However, biomedical concepts are often referred to by various synonyms, and multiple studies have shown the importance of expanding query concepts to their synonyms before sending them to IR systems [10-12]. To further model for domain-specific relevance, such as evidence quality in our case, rerankers are often added to finely rerank the candidates returned by retrieval systems. However, such rerankers are typically based on deep learning, and training them requires a large number of labeled instances [13], which are prohibitively expensive to collect in the biomedical domain. Recent large-scale pretrained language models such as Embeddings from Language Models [14] and Bidirectional Encoder Representations from Transformers (BERT) [15] show significant performance improvement over several natural language processing benchmarks such as General Language Understanding Evaluation [16]. BERT is basically a transformer [17] encoder that is pretrained to predict a randomly masked token in the original input. BERT can be effectively used to rank documents given a specific query [18].

In this work, we propose the PM-Search model that tackles the aforementioned problems of traditional search engines to assist the practice of PM. The PM-Search system has two main components: (1) a baseline retriever using query expansion and keyword matching with the ElasticSearch engine; and (2) an evidence reranker that ranks the initial documents returned by ElasticSearch based on their evidence quality. The reranking uses article features as well as pretrained language models under an expert-in-the-loop active learning strategy, where a biomedical language model BERT for Biomedical Text Mining (BioBERT) [19] is fine-tuned interactively with the experts. Our models participated in the TREC PM 2020 as the ALIBABA team and ranked the highest in the evidence quality assessment: PM-Search achieved standard normalized discounted cumulative gain (NDCG) at rank 30 (NDCG@30) of 47.80% and exponential NDCG@30 of 45.19%, outperforming the second-ranking system by large margins.

In summary, our contributions of this work are three-fold:

1. We present PM-Search, which is an integrated IR system specifically designed to assist precision medicine. PM-Search achieved state-of-the-art performance in the TREC PM Track.
2. We used an expert-in-the-loop active learning strategy based on BioBERT to efficiently derive annotations and improve model performance. To the best of our knowledge, this is the first precision medicine search engine that combines active learning and pretrained language models.
3. We thoroughly analyzed the importance of each system feature with a full set of ablation studies, where we found that the most important features included publication types and active learning. We hope the experiments can provide some insights into the potential future directions of PM search engines.

## Methods

### Data and Materials

The TREC 2020 PM Track provided 40 topics for evaluation. Each topic represented a PM query that contains three key elements of a specific patient population: (1) the disease, that is, the type of cancer; (2) the genetic variant, that is, the gene mutation; and (3) the tentative treatment. The topics were synthetically generated by biomedical experts and several examples are shown in (Table 1). The task used the 2019 PubMed baseline as the official corpus, which contains over 29 million biomedical citations. Each citation is composed of the title, authors, abstract, etc, of the article. For each topic, we denoted its disease as , the genetic variant as and the treatment as . The returned articles were denoted as . Each retrieval result was a query-article pair that contained , , and . We also used the publication type and citation count information extracted in PubMed as additional data sources.

The evaluation of the task followed standard TREC procedures of ad hoc retrieval, where participants submitted a maximum number of 1000 ranked articles and up to 5 different runs for each topic. The assessments were divided into 2 phases, where phase 1 was “Relevance Assessment,” judging the relevance of each article, and phase 2 was “Evidence Assessment,” judging the evidence quality provided by the article.

Phase 1 assessment was a general IR assessment that only considered relevance, where the assessors first judged whether the returned article *a* is generally related to PM. For the PM papers, the assessors then assessed whether the *d*, *g*, and *t* were exact, partially matching, or missing in *a*. Finally, the results were classified as “Definitely Relevant,” “Partially Relevant,” or “Not Relevant” based on a predefined rules of how the *d*, *g*, and *t* matched. The evaluation metrics used in phase 1 include precision at rank 10 (P@10), inferred NDCG (infNDCG), and R-precision (R-prec). P@10 and R-prec are precisions at different ranks:



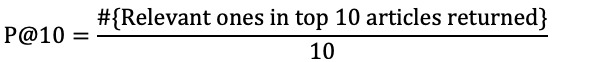





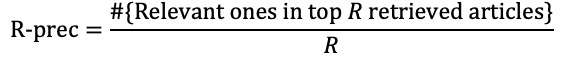



where is the number of relevant articles for the query. NDCG is computed by:



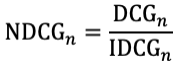



where



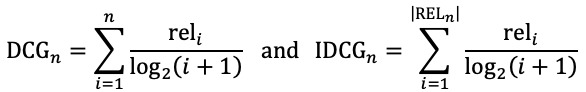



rel*_i_* is the relevance score of article *i* and |REL_n_| denotes the number of relevant articles ordered by the relevance up to position *n*. Since not all submitted articles would be judged by the organizers, there cannot be an exact value of NDCG. To deal with this issue, a sample set of all articles in the top 30 ranks and a 25% sample of articles in ranks 31-100 was used to compute the NDCG, that is, infNDCG.

In the phase 2 assessment, the assessors scored the relevant papers from the phase 1 assessment using a 5-point scale. For example, the tier 4 results should be “randomized controlled trial with >200 patients and single drug, or meta-analysis” and tier 0 should be “Not Relevant” for topic 16. The scale was tailored for each topic to adjust for the differences in the disease, genetic variant, and treatment. The main evaluation metric for phase 2 assessment was NDCG@30. NDCG values at this phase are exact since all articles in the top 30 ranks are judged. Two sets of relevance values were used to compute NDCG, the standard gains (std-gains) and the exponential gains (exp-gains). Standard gains have scores (ie, rel*_i_*) of 0, 1, 2, 3, and 4 corresponding to the 5 tiers, whereas exponential gains have scores of 0, 1, 2, 4, and 8 corresponding to 5 tiers.

**Table 1:** Examples of the Text Retrieval Conference Precision Medicine 2020 topics.

Topic	Disease	Gene	Treatment
1	Colorectal cancer	ABL proto-oncogene 1	Regorafenib
11	Breast cancer	Cyclin dependent kinase 4	Abemaciclib
21	Differentiated thyroid carcinoma	Fibroblast growth factor receptor 2	Lenvatinib
31	Hepatocellular carcinoma	Neurotrophic receptor tyrosine kinase 2	Sorafenib

### PM-Search Overview

**Figure 1:**
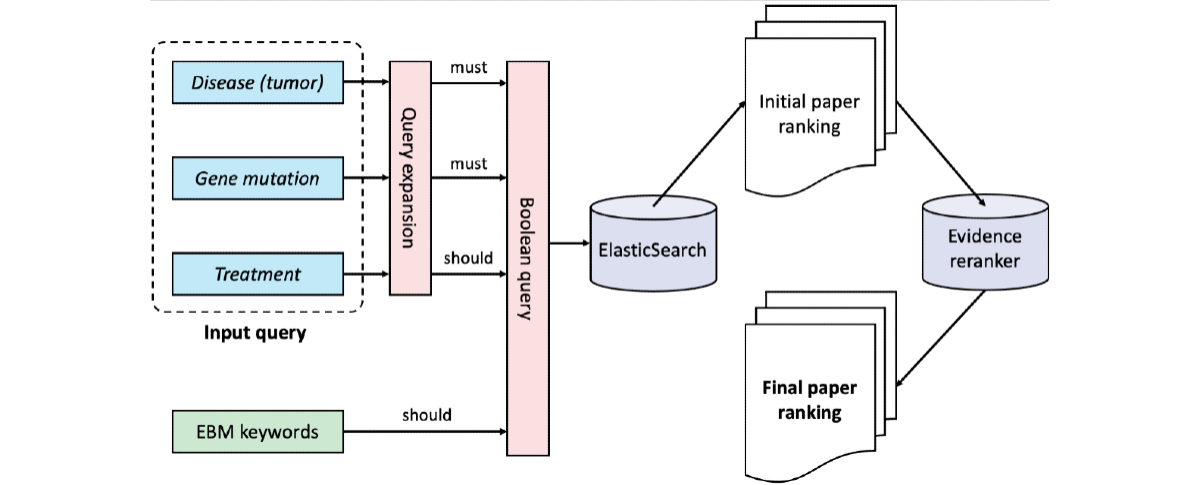
The architecture of PM-Search. EBM: evidence-based medicine; PM: Precision Medicine.

As shown in (Figure 1), PM-Search uses a 2-step approach to retrieve relevant articles for each given PM topic: (1) a *baseline retriever* that is fast and scalable, generating a relatively small number (eg, thousands) of candidates out of millions of PubMed articles—the baseline retriever is based on ElasticSearch (reference) where the original queries are expanded by a list of weighted synonyms; and (2) an *evidence reranker* that finely reranks the retrieved documents based on their evidence quality—the evidence reranker combines the predictions from a BioBERT fine-tuned by an expert-in-the-loop active learning strategy and a feature-based linear regressor.

### Baseline Retriever

We indexed the titles and abstracts of all articles from the PubMed 2019 baseline provided by the TREC organizers using ElasticSearch, a Lucene-based search engine. The synonyms of the disease *d* and gene variant *g* were found via the National Library of Medicine’s web application programming interface in MedlinePlus [20,21]. We denoted the retrieved synonyms of *d* and *g* as {*d*_1_, *d*_2_, ... , *d_m_*} and {*g*_1_, *g*_2_, ... , *g_m_*}, where *d*_1_ = *d* and *g*_1_ = *g*. We did not expand the treatment because the provided term either had no synonym or was used in almost all articles.

For each synonym *d*_1_ and *g*_1_, we counted their document frequency *df*(*d_i_*) and *df*(*g_i_*) in the baseline corpus and calculated the weights of each synonym used in ElasticSearch:



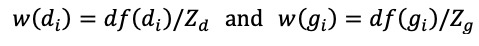



where



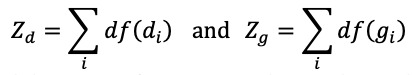



We used the normalized document frequency to lower the ranks of rare terms.

We performed the retrieval in ElasticSearch, which ranks the documents based on their word-level relevance with the input query using the Okapi BM25 algorithm [22]. At the highest level, we queried the ElasticSearch indices using a Boolean query that *must match* the disease and treatment query and *should match* the gene query. The disease, treatment, and gene queries were all *dis_max* queries composed of their synonyms with the weights as boost factors. The *tie_breaker* was set to 0.8 and the title field had a 3.0 boost factor, whereas that of the abstract field was 1.0. In addition, the Boolean query *should match* a list of keywords, including words such as “trial” and “patient” that are chosen empirically to serve as a weak classifier for evidence-based PM papers.

TREC PM allowed a maximum number of 1000 documents per topic in the submission. We set the maximum number of retrieved documents for each topic as 10,000. On average, we retrieved 1589 candidates from the baseline retriever for each topic.

### Evidence Reranker

#### Overview

The Evidence Re-ranker scores a given candidate article *a* based on its evidence quality for the query *q* by:



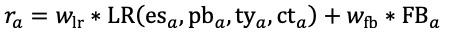



where *r_i_* is the output score, which is a weighted sum of: (1) a linear regressor (LR) using the features of the ElasticSearch score (es), pretrained BioBERT (pb), publication type (ty), and citation count (ct); and (2) a fine-tuned BioBERT (FB). *w*_LR_ and *w*_FB_ are the corresponding weights of the LR and FB. The FB is trained by the expert-in-the-loop active learning strategy, and the LR is trained by expert annotations.

#### Expert-in-the-Loop BioBERT

BioBERT [19] is a biomedical version of BERT that is trained on PubMed abstracts and PubMed Central articles. BioBERT achieves state-of-the-art performance on several biomedical natural language processing tasks. We followed the same setting as Nogueira et al [18] to use BioBERT in this task: to predict the evidence quality of a candidate article *a* for the query *q*, we first feed the concatenated *q* and *a* to the BioBERT, getting the pair representation *h*:



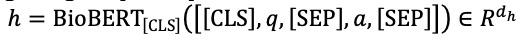



where *q* is the concatenated disease *d*, gene variant *g*, and treatment *t* in the query; *a* is the concatenated title and abstract of the article; and [SEP] is a special token in BERT to mark the input segments. A sigmoid layer is applied to the [CLS] representation *h* to predict the evidence quality 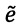:



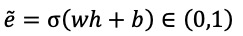



where σ denotes the sigmoid function, *w* and *b* are the layer weights. During fine-tuning, we minimized the mean square loss between the predicted evidence quality 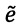 and the expert-labeled score *r*. BioBERT fine-tuning is implemented using Huggingface’s transformers Python package [23]. We use the Adam optimizer [24] with a learning rate of 4 × 10^-5^, batch size of 16, and fine-tuning epoch number of 10 in each iteration.

**Figure 2:**
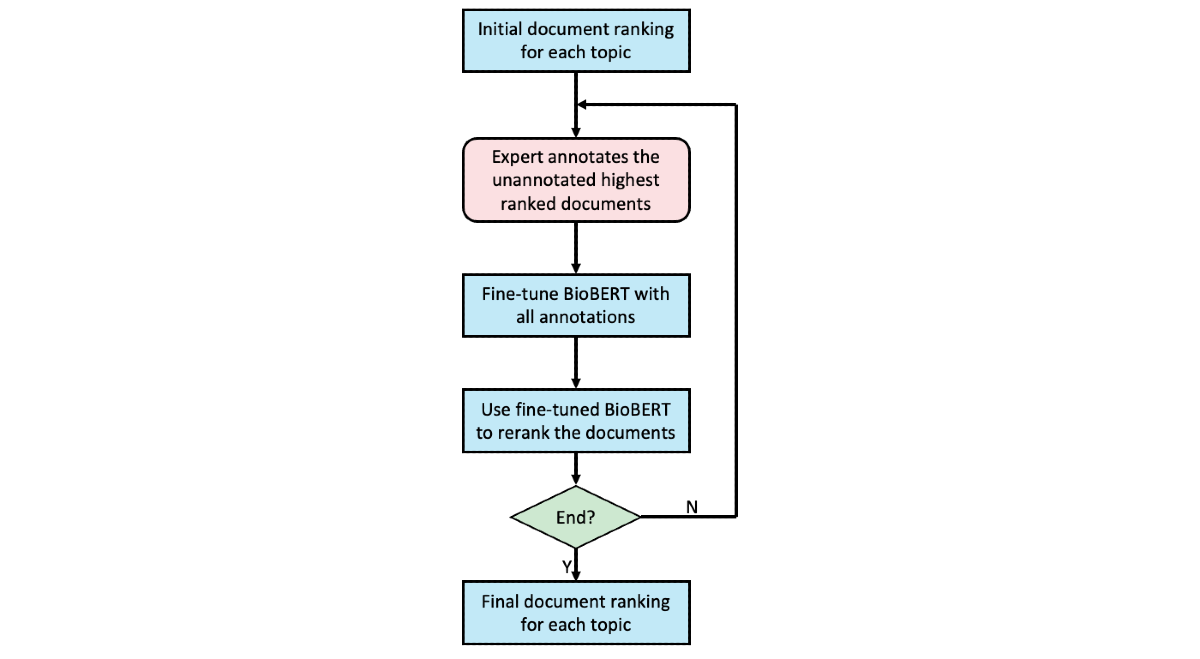
The architecture of our expert-in-the-loop active learning strategy. BioBERT: Bidirectional Encoder Representations from Transformers for Biomedical Text Mining; Y: yes; N: no.

**Figure 3:**
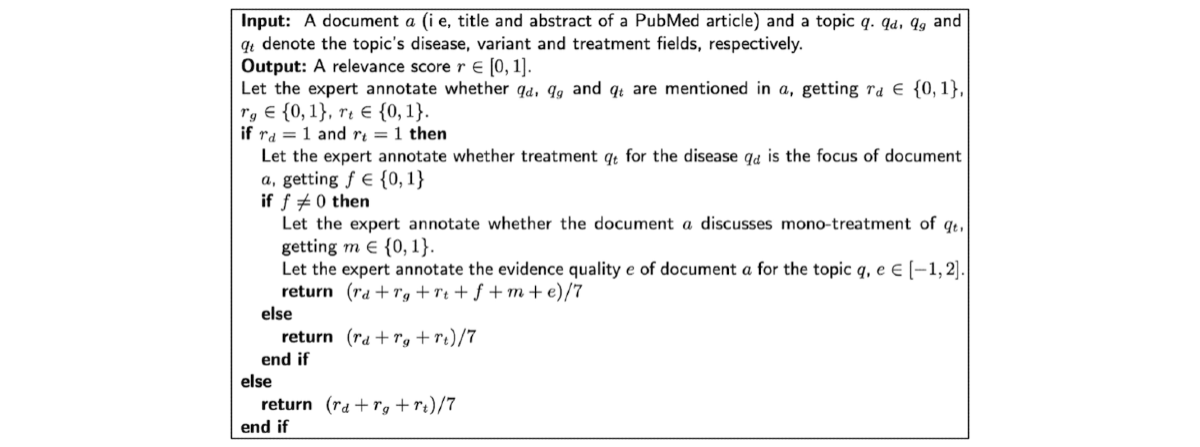
The expert annotation pipeline.

We show the expert-in-the-loop active learning procedure in (Figure 2). At each iteration, a biomedical expert (senior MD candidate) annotates the evidence quality of the highest-ranked unannotated document for the given query based on the criteria shown in (Figure 3). This is similar to the top-1 active feedback setting described in Shen and Zhai [25]. Subsequently, we fine-tuned the original BioBERT with all available annotations at this iteration (ie, the newly annotated instances plus all available annotations from the last iteration) and then used the fine-tuned BioBERT to update the predictions for all documents, leading to the new document rankings. Again, the new document rankings were sent to the expert for annotations. We performed 22 iterations of the expert-in-the-loop active learning, where in most iterations, 40 new annotations were added (1 for each topic), resulting in 950 annotations in total. We also randomly sampled 100 topic-article pairs to be annotated by another medical doctor. The Pearson correlation was 0.853 between the annotation scores of 2 annotators, indicating a high level of interannotator agreement.

#### Linear Regressor

We used the expert annotations to train a simple linear regression model using the following features:

1. es: the relevance scores returned by the ElasticSearch;
2. pb: the relevance scores predicted by a pretrained BioBERT. We used the annotations from the previous TREC PM challenges to fine-tune the BioBERT. Specifically, we collected 54,500 topic-document relevance annotations from the *qrel* files of TREC PM 2017-2019, where the queries contained disease, gene variant, and demographics information but not the treatment option. To ensure consistency, we only used the disease and gene variant fields of the queries as input and fine-tuned the BioBERT to predict their normalized relevance in the annotations. We denoted this as “pretrained” BioBERT since the training data were formatted differently from the data of TREC PM 2020;
3. ty: the publication type score. PubMed also indexes each article with a publication type, such as journal article, review, clinical trials, etc. We manually rated the score of each publication type based on the judgments of their evidence quality. Our publication type and score mapping is shown in Table 2;
4. ct: the citation count score. We ranked the citation count of all PubMed articles and used the quantile of a specific article’s citation count as a feature. Similar to but simpler than PageRank [26], this feature was designed to reflect the community-level importance of each article.

The linear regression was implemented using the *sklearn* Python package, which basically minimizes the residual sum of squares between the expert annotations and the predictions from the linear approximation.

**Table 2:** Mappings between publication types and clinical evidence quality scores.

Publication type	Score
Comment	–1
Editorial	–1
Published erratum	–2
Retraction of publication	–2
English abstract	0
Journal article	0
Letter	0
Review	0
Case reports	1
Observational study	1
Clinical trial	2
Meta-analysis	2
Systematic review	2

### Experiment Settings

We compared our PM-Search submissions to TREC PM 2020 with models submitted by other teams. We used 5 settings in the challenge, namely *PM-Search-auto-1*, *PM-Search-auto-2*, *PM-Search-full-1*, *PM-Search-full-2*, and *PM-Search-full-3*. They use different rerankers to rank the same set of documents retrieved by the baseline retriever. *PM-Search-full-1*, *PM-Search-full-2*, and *PM-Search-full-3* use the evidence reranker. They use the full PM-Search architecture with different combining weights in the evidence reranker.

We also used the *PM-Search-auto-1* and *PM-Search-auto-2* settings that do not use the expert-in-the-loop active learning strategy. Since these settings do not rely on expert annotations, they are considered as the “automatic” runs by the TREC challenge. Specifically, the reranking scores of article *a* for a given query in *PM-Search-auto-1* and *PM-Search-auto-2* are calculated as a weighted sum of the LR features:



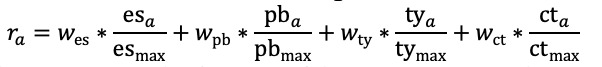



where es*_a_*, pb*_a_*, ty*_a_*, ct*_a_* are the features of document *a*; es_max_, pb_max_, ty_max_, ct_max_ are the corresponding maximum feature values among all documents; and *w*_es_, *w*_pb_, *w*_ty_, and *w*_ct_ are the weights associated with different features and are determined empirically. The feature weights of the submitted systems are shown in Table 3.

**Table 3:** Feature weights in different systems. Participant denotes the system name submitted to the Text Retrieval Conference (TREC) Precision Medicine (PM).

System			TREC run Id		*w*_es_^a^	*w*_pb_^b^		*w*_ty_^c^		*w*_ct_^d^		*w*_LR_^e^		*w*_FB_^f^
**PM-Search runs**														
	PM-Search-auto-1	damoespb1		1.0		0.5	1.5		0.0		—^g^		—	
	PM-Search-auto-2	damoespb2		1.0		0.5	1.0		0.0		—		—	
	PM-Search-full-1	damoespcbh1		–0.465		–0.141	–0.617		–0.005		1.0		1.0	
	PM-Search-full-2	damoespcbh2		–0.465		–0.141	–0.617		–0.005		1.0		2.0	
	PM-Search-full-3	damoespcbh3		–0.465		–0.141	–0.617		–0.005		1.0		5.0	
**Ablations**														
	Retriever + pb	N/A^h^		1.0		1.0	0.0		0.0		—		—	
	Retriever + ty	N/A		1.0		0.0	1.0		0.0		—		—	
	Retriever + ct	N/A		1.0		0.0	0.0		1.0		—		—	
	LR	N/A		–0.465		–0.141	–0.617		–0.005		1.0		0.0	
	FB	N/A		–0.465		–0.141	–0.617		–0.005		0.0		1.0	

^a^es: ElasticSearch score.

^b^pb: pretrained BioBERT.

^c^ty: publication type.

^d^ct: citation count.

^e^LR: linear regressor.

^f^FB: fine-tuned BioBERT (Bidirectional Encoder Representations from Transformers for Biomedical Text Mining).

^g^Not available.

^h^N/A: not applicable.

## Results

### Main Results

The main results of our participating systems in the TREC PM 2020, compared with the other top-ranking systems, are shown in Table 4 [9].

**Table 4:** Topic-wise averaged performance of different settings in the evaluation. All numbers are percentages. Other top-ranking Text Retrieval Conference (TREC) submissions listed in the table include the systems of BIT.UA [27], CSIROMed [28], and h2oloo [29].

			Evidence quality (phase 2)				General relevance (phase 1)		
			NDCG@30^a^, exponential		NDCG@30, standard		infNDCG^b^	P@10^c^	R-prec^d^
**All TREC runs**									
	First	45.19 (ours)		47.80 (ours)		53.25[27]		56.45 [28]	43.58 [28]
	Second	41.93* [29]		42.38* [29]		53.03 [28]		55.16 [27]	42.07 [27]
	Median	28.57		25.29		43.16		46.45	32.59
**PM-Search runs**									
	PM-Search-full-3	45.19		47.80		44.24		47.42	34.72
	PM-Search-full-1	44.97		47.30		43.04		47.42	34.10
	PM-Search-full-2	44.95		47.46		43.84		47.10	34.14
	PM-Search-auto-1	42.55		44.17*		45.33		47.42	35.93
	PM-Search-auto-2	42.54		44.60*		41.12		44.52	32.37
**Ablations**									
	Retriever + pb^e^	32.36*		37.04*		52.26		53.87	41.21
	Retriever + ty^f^	41.46*		43.26*		37.80		40.32	29.37
	Retriever + ct^g^	35.55*		38.40*		42.20		44.84	32.52
	Linear regressor	42.86*		44.86*		37.65		46.13	30.74
	Linear regressor, leave-one-out	42.08*		43.81*		37.06		46.45	30.58
	Fine-tuned BioBERT^h^	44.40*		47.01*		44.59		47.42	34.87
	Fine-tuned BioBERT, leave-one-out	44.15*		46.58*		43.83*		46.45*	33.81*

^a^NDCG@30: normalized discounted cumulative gain NDCG at rank 30.

^b^infNDCG: inferred NDCG.

^c^P@10: precision at rank 10.

^d^R-prec: R-precision.

^e^pb: pretrained BioBERT.

^f^ty: publication type.

^g^ct: citation count.

^h^BioBERT: Bidirectional Encoder Representations from Transformers for Biomedical Text Mining.

*Significant differences from the PM-Search-full-3. Significance is defined as *P*<.05 in 2-sided paired *t* test.

#### General Relevance (Phase 1)

Our submissions scored higher than the topic-wise median submission, but the best submission (infNDCG: 0.5325, P@10: 0.5645, R-prec: 0.4358) outperformed our submissions (infNDCG: 0.4533, P@10: 0.4742, R-prec: 0.3593). Our PM-Search runs (*PM-Search-full-1* to *3*; ie, PM-Search) showed no significant improvements over the runs without active learning (*PM-Search-auto-1* and *2*). It is not surprising, since we focused on modeling evidence quality, and articles that are highly related to the queries but are of low evidence quality (eg, narrative reviews) will be ranked lower. As a result, our submissions performed only moderately in the phase 1 assessment that mainly judges the general relevance.

#### Evidence Quality (Phase 2)

Our PM-Search system *PM-Search-full-3* achieved the highest scores for standard gain NDCG@30 of 0.4780 and exponential gain NDCG@30 of 0.4519. As expected, the *PM-Search-full* settings outperform the *PM-Search-auto* settings that only use the features (0.4503 vs 0.4255 for averaged exponential NDCG@30). This shows that our expert annotation procedure as well as the expert-in-the-loop active learning strategy can improve the performance of evidence quality ranking. Remarkably, all our settings outperform the second-best system (0.4238 for standard NDCG@30 and 0.4193 for exponential NDCG@30) [29], including the *PM-Search-auto* settings that do not rely on expert annotations (exponential NDCG@30: 0.4255). The results show that the proposed PM-Search system is a robust evidence retriever that can be potentially applied to assist the practice of PM.

### Ablations and Feature Importance

We also experimented with different settings and studied the importance of PM-Search components, including the baseline retriever, active learning, and the reranking features.

#### Baseline Retriever Settings

In Table 5, we show the performance of the baseline retriever without query expansion or keyword matching. The results show that query expansion is an important module to improve the recall of relevant articles. However, we find that boosting keywords such as “trial” and “patient” do not significantly change the performance. This is inconsistent with the study of Faessler et al [10], which shows that boosting a range of keywords helps improve the performance. One key difference between our system and Faessler et al [10] is that we only use 2 positive keywords, whereas they use various positive and negative keywords, so increasing the number and diversity of keywords could be a future work for improvements.

**Table 5:** Ablation results of different baseline retriever settings (in percentages).

Method	Evidence quality (phase 2)				General relevance (phase 1)		
	R@0.5k^a^	R@1k^b^	R@10k^c^	R@0.5k		R@1k	R@10k
Baseline retriever	68.99	75.96	81.00	65.51		72.30	77.71
Baseline retriever without query expansion	66.84*	72.61*	76.94*	61.85*		67.21*	72.90*
Baseline retriever without keyword matching	68.85	76.06	81.00	65.65		72.33	77.71

^a^R@0.5k: recall at the top 500 positions.

^b^R@1k: recall at the top 1000 positions.

^c^R@10k: recall at the top 10,000 positions.

*Significant differences than the original retrieval. Significance is defined as *P*<.05 in 2-sided paired *t* test.

#### Active Learning

**Figure 4:**
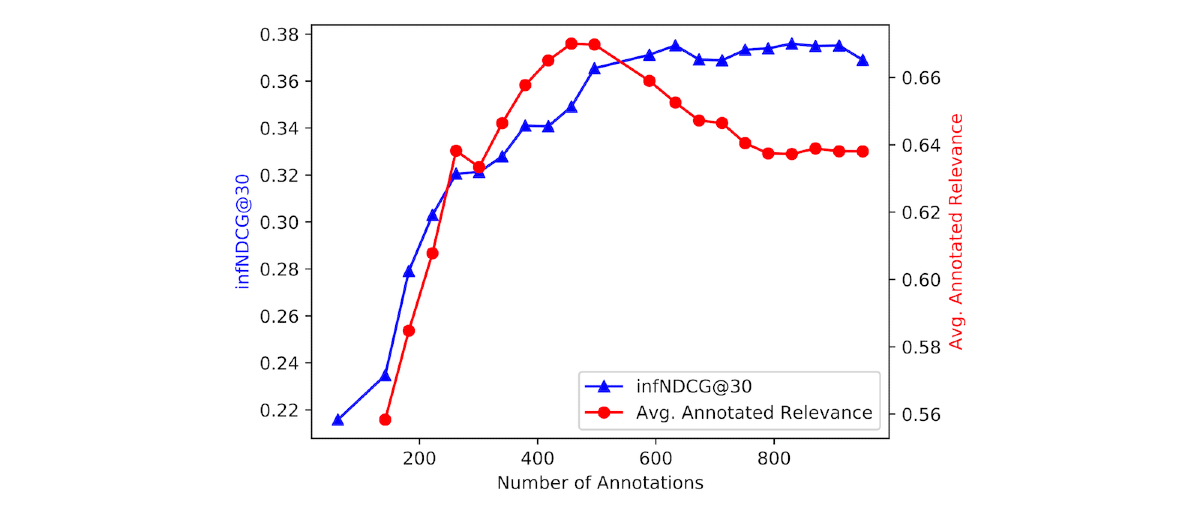
InfNDCG@30 and average annotated relevance at each iteration in active learning. InfNDCG@30: inferred normalized discounted cumulative gain at rank 30.

In Figure 4, we show the performance of the BioBERT predictions at each iteration in active learning, evaluated with infNDCG@30 by the evidence quality (phase 2) assessments. The performance increases with the iteration when the number of annotations is less than 500 and then converges after the number of annotations is greater than 500. Interestingly, we find that the average annotated relevance by our annotator also reaches its maximum at around 500 annotations, which indicates that this metric can be empirically used as the stop criterion.

#### Reranker Features

To analyze the importance of the used features, we show the ablation experiments in Table 4 and Pearson correlations between them and the official scores in both phases in Table 6.

General relevance (phase 1): BioBERT that is further pretrained by the annotations of previous TREC PM (pb) had the highest correlation (0.5771) with the phase 1 scores, and the baseline retriever with the pretrained BioBERT had the highest performance (infNDCG: 52.26%) in our ablation experiments. This is probably because the evaluations of previous tasks are also based on general relevance. The ElasticSearch scores (es) achieved the second highest correlation of 0.3892, and the fine-tuned BioBERT by active learning (FB) had a Pearson correlation of 0.3733. However, our expert annotations for the evidence quality only had a Pearson correlation of 0.2157 with the general relevance scores, which indicates that generally relevant papers might not have high evidence quality. In addition, the features of publication types (ty) and the citation counts (ct), which are designed for the evidence quality ranking and are positively correlated with the evidence quality, were negatively correlated with the general relevance scores.

Evidence quality (phase 2): The trends of ablation results and correlations between features and evidence quality scores were similar in both the standard and exponential scores. The most important features in the evidence quality evaluation included publication types and active learning. Interestingly, only using the publication type and the baseline retriever achieves comparable performance to the second-best system in TREC PM (0.4146 vs 0.4193 for exponential NDCG@30). BioBERT fine-tuned by the expert annotations (FB) had the highest performance in the ablation experiments (exponential NDCG@30: 0.4440) and its correlation to the official annotations was close to that of our expert annotations (0.3309 vs 0.2937 for exponential gains; 0.2847 vs 0.3073 for standard gains). Besides, the fine-tuned BioBERT outperformed the expert annotations by a large margin (0.3733 vs 0.2157) in the phase 1 assessment, indicating that it can rerank the documents by evidence quality while retaining the original general relevance ranks to some extent. The most correlated features of phase 1, that is, the pretrained BioBERT (pb) and the ElasticSearch score (es), had the lowest correlations with the phase 2 scores, which further confirms that the evidence quality assessment is distinct from the general relevance assessment.

In summary, the 2 assessment phases might have opposite considerations since features that are highly related to the score of one phase tended to be much less related to the score of the other phase, with the exception of the fine-tuned BioBERT. As a result, specific modeling of evidence quality beyond general relevance is required for a PM search engine.

**Table 6:** Feature correlations to the official scores.

Features			es^a^		pb^b^	ty^c^		ct^d^		LR^e^	FB^f^		Expert annotation
General relevance			0.3892		*0.5771*	–0.0621		–0.0435		0.1341	0.3733		0.2157
**Evidence quality**													
	Standard gains	0.0752		0.0621		0.2564	0.0696		0.2728		*0.3309*	0.2937	
	Exponential gains	0.0474		0.0338		0.2772	0.0806		0.2816		0.2847	*0.3073*	

^a^es: ElasticSearch score.

^b^pb: pretrained Bidirectional Encoder Representations from Transformers for Biomedical Text Mining (BioBERT).

^c^ty: publication type.

^d^ct: citation count.

^e^LR: linear regressor.

^f^FB: fine-tuned BioBERT.

## Discussion

### Topic-Level Generalizability Analysis

Each instance used to train the PM-Search reranker contained a topic-article pair and its relevance score. The main results show that PM-Search is generalizable at *instance-level*, where the model is trained and evaluated by different instances. However, *topic-level* generalizability of the PM-Search was not evaluated since our expert annotations and the official annotations, that is, the training and evaluation instances, used the same set of topics.

Here, we analyze how PM-Search generalizes to unseen topics using a leave-one-out evaluation strategy. Each time, we use the official annotations of only one topic to evaluate the models that are trained by our expert annotations without the evaluation topic. The results of each topic as the evaluation topic are calculated and the averaged performance is shown in Table 4. The leave-one-out results are close to the results when all expert annotations are used for training: 0.4415 versus 0.4440 for exponential NDCG@30 and 0.4658 versus 0.4710 for standard NDCG@30. This shows that the model is also generalizable to unseen topics.

### Error Analysis

We show several typical cases in Table 7 to qualitatively analyze some errors in the evidence quality assessment. It should be noted that most errors cannot be attributed to a specific cause since the predictions of BioBERT are not explainable, so developing explainable models is a vital future direction to explore.

**Table 7:** Typical error cases in the evidence quality assessment. Topics are shown in Table 1.

Case	Topic	Article	Official, rank (normalized relevance)	PM^a^-Search, rank (normalized relevance)	Error type
1	1	PMID^b^: 23177515; Title: Efficacy and safety of regorafenib for advanced gastrointestinal stromal tumours after failure of imatinib and sunitinib (GRID): an international, multicentre, randomised, placebo-controlled, phase 3 trial	1 (1.00)	N/A^c^	Concept recognition
2	1	PMID: 24150533; Title: Risk of hypertension with regorafenib in cancer patients: a systematic review and meta-analysis	1 (1.00)	148 (0.47)	Different understanding
3	1	PMID: 25213161; Title: Randomized phase III trial of regorafenib in metastatic colorectal cancer: analysis of the CORRECT Japanese and non-Japanese subpopulations	1 (1.00)	297 (0.29)	Unclassified
4	11	PMID: 29147869; Title: Hematological adverse effects in breast cancer patients treated with cyclin-dependent kinase 4 and 6 inhibitors: a systematic review and meta-analysis	1 (1.00)	N/A	Full article visibility
5	11	PMID: 28540640; Title: A Population Pharmacokinetic and Pharmacodynamic Analysis of Abemaciclib in a Phase I Clinical Trial in Cancer Patients	1 (1.00)	53 (0.50)	Full article visibility
6	11	PMID: 29700711; Title: Cyclin-dependent kinase 4/6 inhibitors in hormone receptor-positive early breast cancer: preliminary results and ongoing studies	61 (0.25)	6 (0.71)	Different understanding

^a^PM: precision medicine.

^b^PMID: PubMed IDentifier.

^c^N/A: not applicable.

#### Full Article Visibility

The PM-Search system can only access the title and abstract of PubMed articles. However, vital article information (eg, detailed gene variant types, treatments) might only appear in the full article, especially for meta-analyses and systematic reviews where abstracts tend to use more general concepts. For example, PM-Search fails to retrieve the Case 5 article where the queried disease “breast cancer” is only mentioned in the full article, not in the abstract. For this, future models can use the full article information from PubMed Central to better retrieve and rank relevant papers.

#### Different Understanding

In some cases, we have a different understanding of how clinically significant the evidence is that an article provides. For example, the article “Risk of hypertension with regorafenib in cancer patients: a systematic review and meta-analysis” in Case 2 is focused on the hypertension side effect of the therapy, not the therapeutic effects, which we think is not significant. However, it was given the highest score in the official evaluation but ranked much lower in the PM-Search prediction. This issue should be solved by community efforts for the development of standards.

#### Concept Recognition

The baseline retriever of PM-Search uses query expansion to recognize relevant concepts in the article. However, this step is error prone since biomedical terms are highly variable and thus cannot be represented by a list of synonyms. For example, in Case 1, the “colorectal cancer” in the query appears as “gastrointestinal stromal tumours” in the article, which was missed in the query expansion step of PM-Search. As a result, this article was not returned by the PM-Search but ranked the highest in the official assessment. Improving similar concept recognition, such as using distributed representations of concepts, remains an important direction to explore.

### Comparison With Prior Work

Many IR systems for precision medicine have been proposed in the TREC PM tracks [7-9,30], where the key issue to solve is that queries and their related documents might use different terms to describe the same concepts. Some studies [31-33] have attempted to use BERT-based models for ranking in previous TREC PM tracks, showing various levels of improvements. Thalia is a semantic search engine for biomedical abstracts that is updated on a daily basis [34]. It tackles the vocabulary mismatch problem by mapping the queries to predefined concepts by which the documents are indexed. The HPI-DHC team shows that query expansion associated with hand-crafted rules improves the retrieval performance [35]. Faessler et al [10,36] systematically analyze the individual contributions of relevant system features such as BM25 weights, query expansion, and boosting settings. PRIMROSE is a PM search engine that expands the queries with an internal knowledge graph [37]. Noh and Kavuluru [38] use a basic BERT with specific components for reranking. Koopman et al [39] present a search engine for clinicians to find tailored treatments for children with cancer. For the vocabulary mismatch issue, PM-Search uses a similar query expansion strategy to previous studies. However, PM-Search differs from all prior work in that it is specifically designed to rank the retrieval results by their evidence quality, which is an important feature for PM search engines.

### Conclusions and Future Work

In this paper, we present PM-Search, a search engine for PM that achieved state-of-the-art performance in TREC PM 2020. PM-Search uses an ElasticSearch-based baseline retriever with query expansion and keyword matching and an evidence reranker that uses the BioBERT fine-tuned by an active learning strategy. Our analyses show that the evidence quality is a distinct aspect from the general relevance, and thus, specific modeling of it is necessary to assist the practices for evidence-based PM.

The deployment and evaluation of PM-Search in real clinical settings remains a clear future direction. It is also worth exploring the use of dense vectors for baseline retrieval and incorporating full-text information into the ranking process.

## Abbreviations

BERT: Bidirectional Encoder Representations from Transformers
BioBERT: Bidirectional Encoder Representations from Transformers for Biomedical Text Mining
BNDCG@30: normalized discounted cumulative gain NDCG at rank 30
ct: citation count
es: ElasticSearch score
FB: fine-tuned BioBERT
infNDCG: inferred normalized discounted cumulative gain
IR: information retrieval
LR: linear regressor
NDCG: normalized discounted cumulative gain
NDCG@30: NDCG at rank 30
P@10: precision at rank 10
pb: pretrained BioBERT
PM: precision medicine
R-prec: R-precision
TREC: Text Retrieval Conference
ty: publication type
